# Ly6C^hi^ inflammatory monocytes promote susceptibility to *Leishmania donovani* infection

**DOI:** 10.1038/s41598-017-14935-3

**Published:** 2017-10-31

**Authors:** Cesar Terrazas, Sanjay Varikuti, Steve Oghumu, Heidi M. Steinkamp, Nurittin Ardic, Jennifer Kimble, Hira Nakhasi, Abhay R. Satoskar

**Affiliations:** 1 Department of Pathology, The Ohio State University Medical Center, Columbus, OH 43210 USA; 2 Department of Environmental Health Sciences, College of Public Health, The Ohio State University, Columbus, OH USA; 3 Division of Pediatric Dentistry, College of Dentistry, The Ohio State University, Columbus, OH USA; 4 Division of Emerging and Transfusion Transmitted Disease, Center for Biologics Evaluation and Research, Food and Drug Administration, Silver Spring, Maryland USA; 5 Department of Medical Microbiology, Faculty of Medicine, Baskent University, Ankara, Turkey; 6 Departments of Microbiology, Wexner Medical Center, The Ohio State University, Columbus, OH 43210 USA

## Abstract

Ly6C^hi^ inflammatory monocytes (iMO) are critical for host defense against toxoplasmosis and malaria but their role in leishmaniasis is unclear. In this study, we report a detrimental role of Ly6C^hi^ iMOs in visceral leishmaniasis (VL) caused by *Leishmania donovani*. We demonstrate that Ly6C^hi^ iMOs are continuously recruited into the spleen and liver during *L. donovani* infection and they are preferential targets for the parasite. Using microarray-based gene expression profiling, we show that Ly6C^hi^ iMOs isolated from the infected liver and spleen have distinct phenotypic and activation profiles. Furthermore, we demonstrate that blocking the recruitment of Ly6C^hi^ iMOs into the liver and spleen during *L. donovani* infection using a CCR2 antagonist reduces the frequency of the pathogenic IFN-γ/IL10 dual producer CD4+ T cells in the spleen and leads to a significant reduction in parasite loads in the liver and spleen. Using STAT1−/− mice we show that STAT1 is critical for mediating the recruitment of Ly6C^hi^ iMOs into organs during *L. donovani* infection, and adaptive transfer of wild type Ly6C^hi^ iMOs into STAT1−/− recipients renders them susceptible to disease. Our findings reveal an unexpected pathogenic role for Ly6C^hi^ iMOs in promoting parasite survival in VL and open the possibility of targeting this population for host-directed therapy during VL.

## Introduction

Visceral leishmaniasis (VL) is the second leading cause of mortality among tropical diseases. It is caused by *Leishmania donovani* in Asia and Africa and *L. infantum* in South America and the Mediterranean basin. *L. donovani* is an obligate intracellular protozoan that infects professional phagocytes such as neutrophils, macrophages, dendritic cells, and spreads systemically infecting the spleen, liver and bone marrow^1^. As of yet, there is no vaccine available, and current drug treatments have adverse side effects, such as liver and pancreatic damage, and in some cases, death. Investigating the mechanisms that *L. donovani* exploits in order to survive in the host is highly relevant so as to understand disease progression and design new therapies.

It is well established that protective immunity against VL involves a Th1-associated response characterized by the IL12-IFNγ-iNOS pathway, which is necessary for optimal macrophage activation and formation of granulomas^2,3^. STAT1 is an important transcription factor that mediates IFNγ signaling and is central to the development of a Th1 immune response. Surprisingly, mice that are genetically deficient in STAT1, although unable to mount a Th1 immune response, are highly resistant to VL^4^. Mechanisms surrounding STAT1 mediated susceptibility to VL caused by *L. donovani* have remained incompletely understood, but previous studies suggest that T cell independent mechanisms are involved. Understanding the cellular mechanisms surrounding the resistance of STAT1−/− mice to VL will provide insights into *L. donovani* pathogenesis in the host and provide new strategies for treatment against VL.

At the cellular level, *L. donovani* preferentially infects Kupffer cells in the liver and marginal zone macrophages in the spleen^5^. Newly recruited macrophages which are derived from circulating monocytes are also potential host cells and play a crucial role in the establishment of *L. donovani* infection. These circulating monocytes are divided into two subsets: patrolling monocytes (characterized as CD11b+ CX3CR1^lo^ Ly6C^low^ CCR2- CD115+) and inflammatory monocytes iMOs (characterized as CD11b+ CX3CR1^lo^ Ly6C^hi^ CCR2+ CD115+)^6^. iMOs exit the bone marrow in a CCR2 dependent manner, and once in circulation, they infiltrate the tissue^3^. iMOs are activated after pathogen recognition through Toll like receptors (TLRs) and/or IFNγ signaling and produce nitric oxide as well as inflammatory cytokines such as TNFα. Finally, iMOs differentiate into macrophages or dendritic cells (TipDCs) with enhanced microbicidal activity and are indispensable for resistance to a variety of pathogens^7^. Since the activation and subsequent differentiation of iMOs depends in part on STAT1 mediated IFNγ signaling, it is essential that we understand the role of this immune cell subset during infection with *L. donovani*.

iMOs play an important role in immunity against intracellular pathogens^8^. They have been shown to drive strong Th1 immune responses and protection against murine *Toxoplasma* infection^9,10^. Further, blockade of iMOs migration from the bone marrow to infected sites results in increased susceptibility to *Listeria monocytogenes* in mice^3^. iMOs are associated with protection against *Plasmodium vivax* ^11^, although they also contribute to brain pathology in a mouse model of experimental cerebral malaria. In *Leishmania major* infection, iMOs have also been shown to play a protective role^12^. In this study, we investigated the role of iMOs during VL. In contrast to most intracellular pathogens, our results reveal a detrimental role for this immune cell subset. Our results also provide an explanation for the previously observed surprising resistance to *L. donovani* infection that is observed in STAT1−/− mice.

## Results

### Dynamics of inflammatory monocyte recruitment during VL

**Figure 1:**
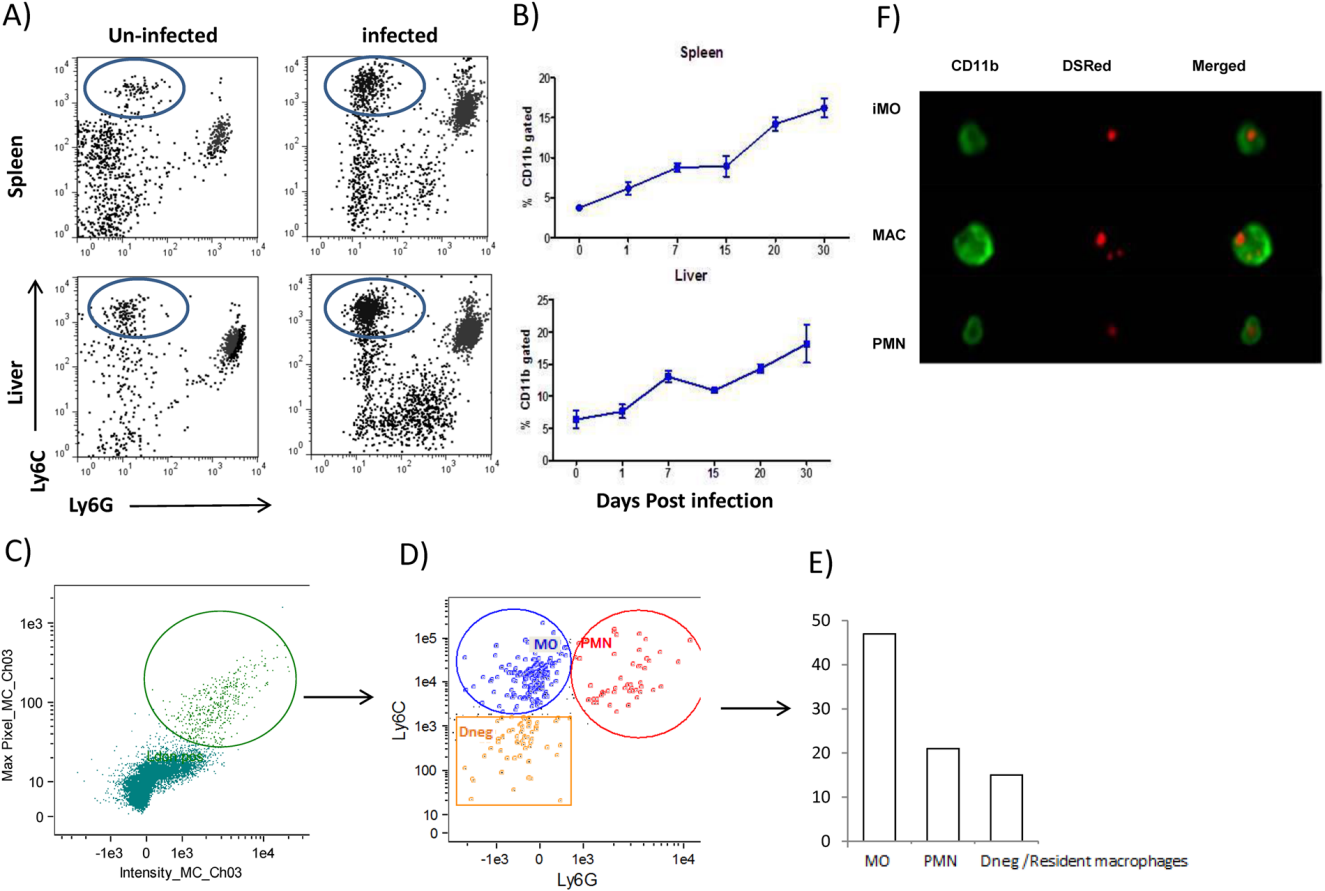
*Leishmania donovani* infection induced preferential recruitment of inflammatory monocytes. (**A**) Flow cytometric analysis of CD11b+ gated cells from the spleen and liver of *L. donovani* infected mice at 24 hr post infection. (**B**) Kinetics of inflammatory monocyte recruitment was evaluated by flow cytometry at the indicated time points in spleen and liver. Data represents the mean ± SEM from 1 of 2 independent experiments (n = 5/time point). *L donovani*-DsRED parasites were inoculated i.p. into WT mice, 24 h later peritoneal exudates were evaluated by Imaging-flow cytometry. Infected cells were gated as shown in (**C**) and analyzed for Ly6C and Ly6G expression (**D**). (**E**) Percentages of different subsets of infected cells, MO: Ly6C^hi^ Ly6G-CD11b+, PMN: Ly6C+ Ly6G+ CD11b+, and Dneg: Ly6C-Ly6G-CD11b^hi+^ (n = 3). (**F**) Representative pictures of the different subsets infected with *L. donovani* analyzed by AMNIS Flow sight.

A variety of infections cause the recruitment of iMOs into infected tissues which results in pathogen clearance^13,14^. During murine VL, monocytes are recruited into the liver resulting in the formation of granulomas during early and chronic phase of the infection. However, what is not known is the kinetics of such monocyte recruitment, and whether the infiltrate consists of Ly6C^hi^ iMOs. In order to investigate the early phase of the immune response during VL, we studied the mobilization of iMOs into *L. donovani* infected organs. To this end, we identified iMOs as CD11b+ Ly6C^hi^ Ly6G^−^ by flow cytometry. The proportion of iMOs increased after 24h post infection (pi) in the liver and spleen (Fig. 1A). Next, we investigated the kinetics of iMO recruitment. We found constant accumulation of iMOs in the liver and spleen over time reaching up to 15–25% of myeloid cells in both organs after 30 days of post infection (dpi) (Fig. 1B). We next investigated the ability of iMOs to phagocytize *L. donovani* parasites. Using transgenic DsRED–*L. donovani* parasites, we looked for infected cells after 24h of inoculation in the peritoneal cavity. We found three subsets infected with *L. donovani*. The cells harboring more parasites were Ly6C^hi^ Ly6G^−^CD11b+. The second population of infected cells consisted of Ly6C+ Ly6G+ CD11b+ neutrophils. Interestingly, resident Ly6C-Ly6G-CD11b+ macrophages were also infected, however they represented the least infected cells among the three populations (Fig. 1C–F). Taken together, *L. donovani* infection resulted in early and continuous recruitment of iMOs into liver and spleen with high capability to uptake *Leishmania* parasites.

### Characterization of inflammatory monocytes recruited during *L. donovani* infection

**Figure 2:**
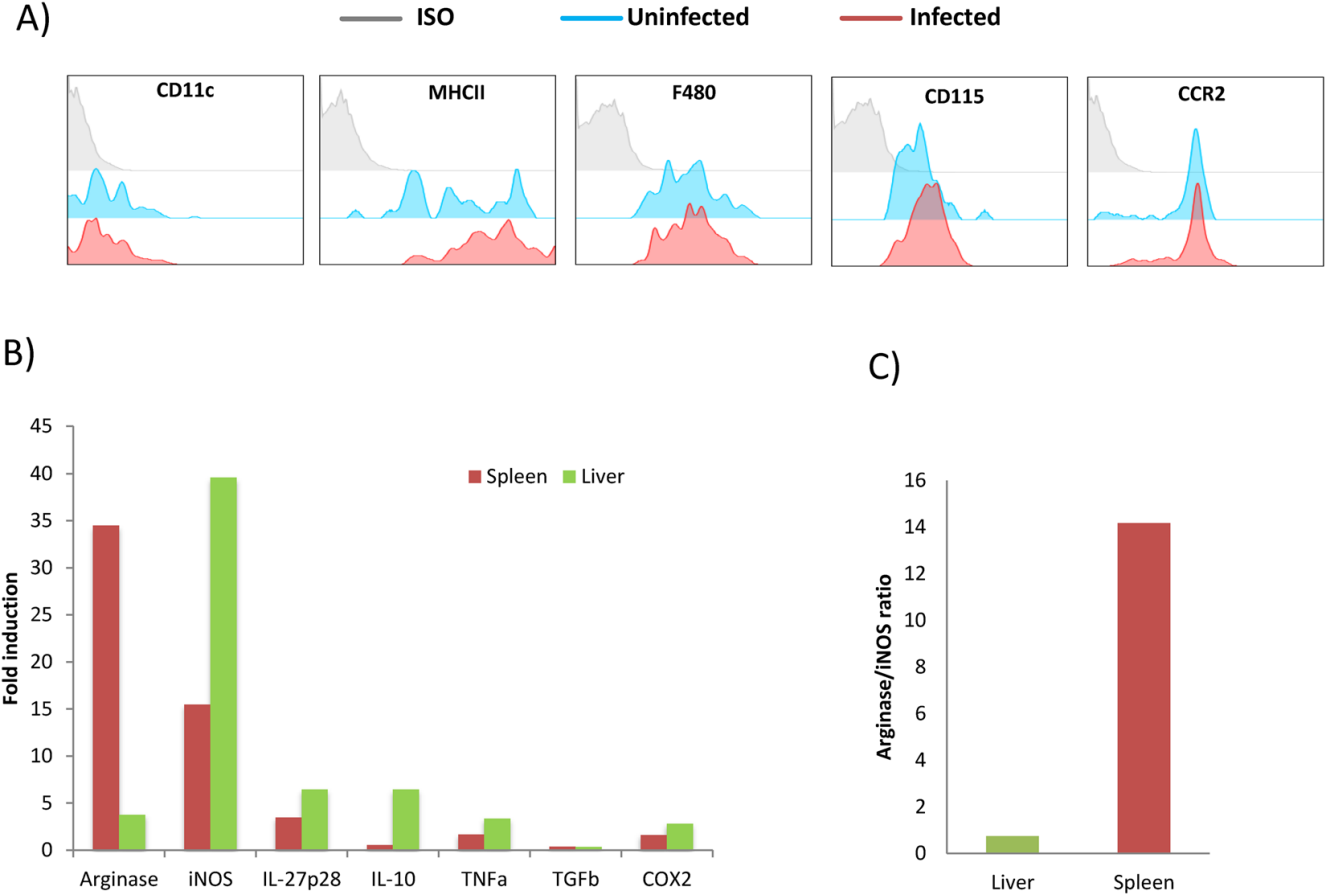
Characterization of inflammatory monocytes phenotype during *Leishmania donovani infection*. (**A**) Flow cytometric analysis of Ly6C^hi^Ly6G-CD11b+ cells from uninfected and *L. donovani* infected WT mice 30 days post infection. Cells were gated on CD11b+ and analyzed for monocyte/macrophages/DC associated markers. Representative overlaid histograms plots were shown. (**B**) Gene expression analysis of sorted Ly6C^hi^Ly6G-CD11b+ cells from spleen and liver of *L. donovani* infected mice. Data represents fold change relative to sorted cells from uninfected mice. Bar graphs show the average gene expression of pooled Ly6C^hi^Ly6G-CD11b+ cells sorted from infected mice (n = 4). (**C**) Gene expression ratio of Arginase:iNOS from Ly6C^hi^ Ly6G- CD11b+ cells sorted from livers and spleens.

In order to confirm the identity of CD11b+ Ly6C^hi^Ly6G- cells observed during *L. donovani* infection, we examined the expression of CD115 (MCSF receptor) and the hallmark chemokine receptor CCR2^3^. These cells expressed CD115 and CCR2 on their cell surface, which is in agreement with the phenotype described for inflammatory monocytes^15^, (Fig. 2A). In some infections, inflammatory monocytes gain CD11c expression and are known as TipDCs^16,17^. However, during chronic infection with *L. donovani*, iMOs did not upregulate CD11c, but expressed intermediate levels of MHCII and F4/80 on their membrane (Fig. 2A), which is suggestive of their differentiation into the macrophage lineage. iMOs are known to express high levels of iNOS in several infections, and nitric oxide production is important in *L. donovani* resistance^18^. Interestingly iNOS expression was higher in liver than in the spleen after 30 dpi, and a similar trend was found for TNFα expression. Unlike iNOS, arginase is associated with *Leishmania* survival inside macrophages^19^. iMOs isolated from the spleen at 30 dpi showed higher arginase expression compared to liver isolated iMOs (Fig. 2B). Interestingly, arginase/iNOS ratio was higher in the spleen where the infection turns progressive, and the opposite trend was found in the liver where the infection starts resolving after 30 dpi (Fig. 2C). It appears from these data that, unlike the liver, the splenic microenvironment causes differentiation of iMOs into a phenotype that favors *Leishmania* survival. Recently, it was reported that iMOs can produce PGE2 depending on the gut microbiota^20^. COX2, an upstream regulator of PGE2 production was slightly overexpressed by iMOs in both the liver and the spleen (Fig. 2B)

### Inflammatory monocytes isolated from spleen and liver during VL present differential activation state

**Figure 3:**
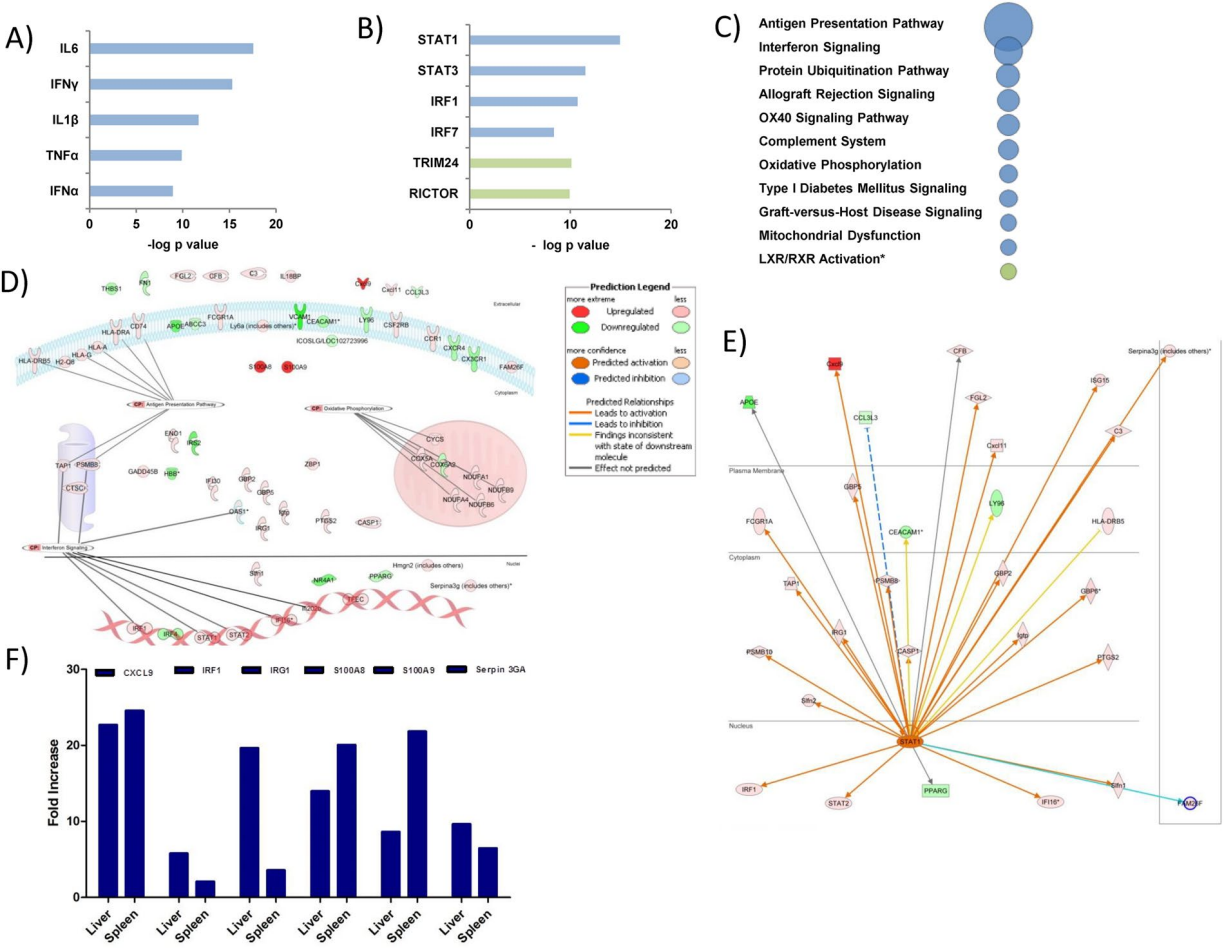
STAT1 is a key molecule in iMOs phenotype. Ly6C^hi^ Ly6G-CD11b+ cells were sorted from the pooled spleen suspensions of naïve or 30 dpi mice (n = 4). RNA obtained from sorted cells was analyzed in Affymetrix microarray chip. Data was analyzed with Ingenuity pathways analysis software, and upstream regulators and networks were constructed. (**A**) Top upstream cytokine regulators on infected iMOs. (**B**) Top transcription factors found by IPA analysis. (**C**) Top canonical pathways associated with iMOs infection. (**D**) Network of genes involved in top 10 upstream regulators. (**E**) STAT1 network in infected iMOs. (**F**) Validation of up regulated genes by RT-PCR, represented fold increase is compared to WT un-infected controls. Bar graphs show the average gene expression of pooled Ly6C^hi^Ly6G-CD11b+ cells sorted from infected mice (n = 4).

The uncommon arginase expression in monocytes isolated from spleen (Fig. 2C) lead us to expand the characterization of those iMOs by using microarray based gene expression profiling. Among the top upregulated genes, we found S100A9, S100A8 and CXCL9 (Fig. 3F). S100A8/9 functions as a chemoattractant for monocytes and neutrophils, while CXCL9 attracts CXCR3 expressing cells such as T and NK cells. These chemokines are enhanced with IFN stimulation^21^. Also, we found upregulation of IFN signaling pathway by increased expression of IRF1 and IGR1. Interestingly, when we compared IRF1 and IRG1 we found higher expression in the liver than in the spleen, which is opposite compared with S100A8/9 expression (Fig. 3F). Further, pathway analyses using Ingenuity Pathway Analysis software revealed other characteristics of iMOs, such as up regulation of antigen presentation associated genes and interferon signaling and complement activation in splenic iMOs after infection (Fig. 3C). Interestingly, splenic iMOs showed enhanced ubiquitination pathway protein expression and enhanced expression of oxidative phosphorylation which has been associated with M2 macrophage metabolism (Fig. 3C,D)^22^, which is in line with our observation of splenic iMOs expressing elevated arginase transcripts (Fig. 2B). Upstream analysis revealed a role for IFNs, STAT1, TLRs, IL1B and IL-6 as main regulators for iMOs gene expression (Fig. 3A,B). To identify key molecules involved in iMO phenotype, we generated a pathway for iMO, interestingly; STAT1 pointed was a key player in iMOs phenotype (Fig. 3E).

### STAT1 is necessary for iMO accumulation in spleen and liver during *L. donovani* infection

**Figure 4:**
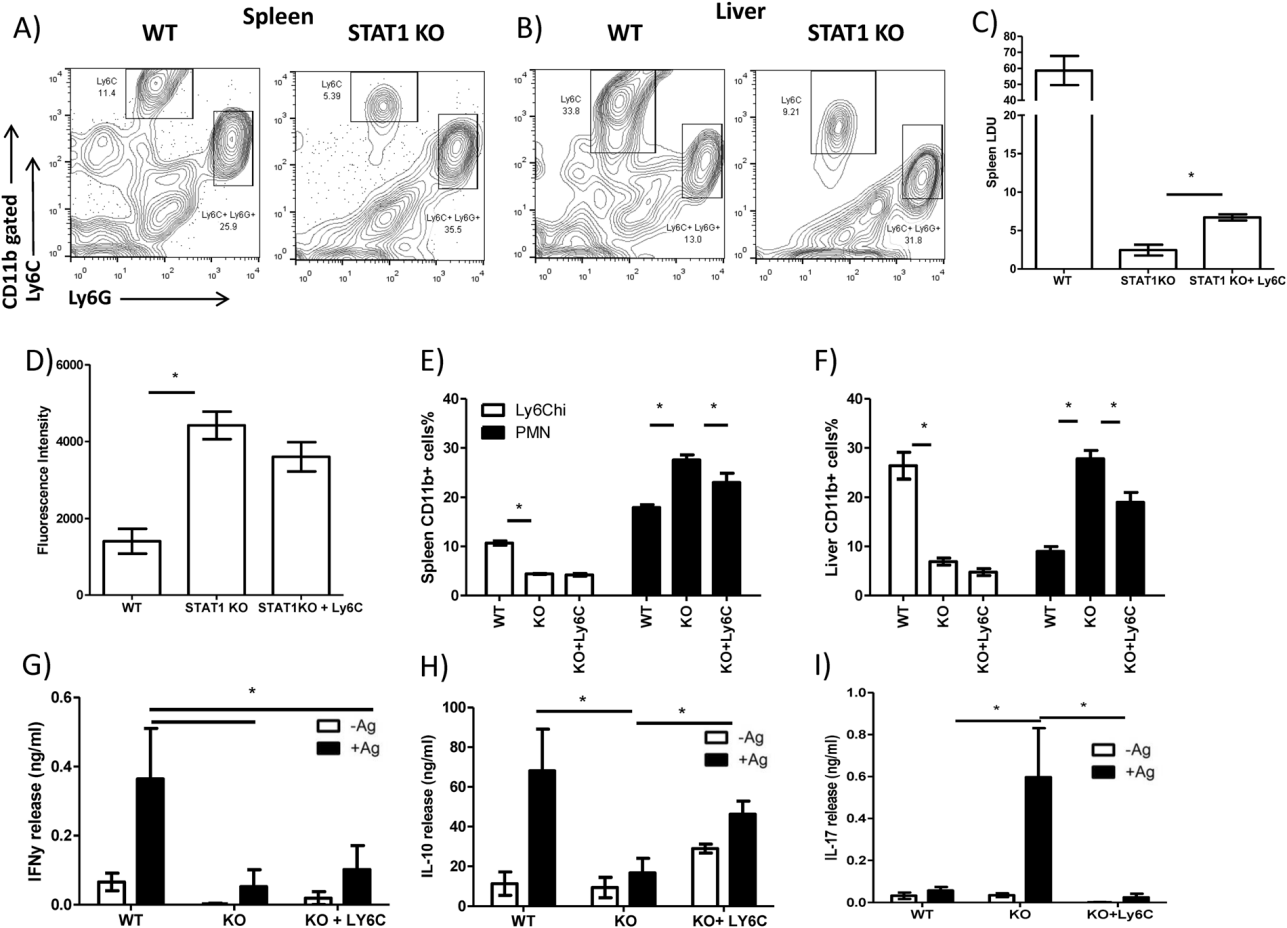
STAT1 plays a critical role in iMO accumulation *in L. donovani* infected tissues. (**A**) Flow cytometric analysis of Ly6C^hi^ cells from the spleen of *L. donovani* infected WT and STAT1KO mice at 20 dpi. (**B**) Flow cytometric analysis of Ly6C^hi^ cells from the liver of *L. donovani* infected WT and STAT1KO mice at 20 dpi. (**C**) Bone marrow iMOS were sorted from naïve WT mice and transferred into *L. donovani* infected STAT1KO mice at 24 h pi, Parasitic loads were analyzed at 20 dpi. (**D**) Splenocytes were re-stimulated with *L. donovani* antigen for 72 h, proliferation was avaluated by using Alamar Blue. (**E**,**F**) Percentages of Ly6C^hi^ (Ly6C^hi^ Ly6G- CD11b+), Ly6C^low^ (Ly6C^low^ Ly6G-CD11b+) and PMN (Ly6C+ Ly6G+ CD11b+) in spleen and liver respectively. (**G**–**I**) Supernatants from antigen re-stimulated splenocytes were evaluated for IFNγ, IL-10 and IL-17 production by ELISA. Data represented from 1 of 3 independent experiments. n = 5, *p < 0.05.

The central roles of STAT1 in the genome profile of iMOs lead us to investigate them in STAT1KO mice. Previously we found that STAT1 KO mice were highly resistant to VL in a T cell independent fashion^4^. Some studies have shown that IFNs could be important for monocyte differentiation in an IRF dependent pathway^23^. The main IFNγ intracellular signaling adaptor is STAT1. However, the role STAT1 plays in monocyte accumulation is unknown. We first investigated the accumulation of iMOs in STAT1 KO mice. As showed before (Fig. 1A) WT mice had enhanced accumulation of CD11b+ Ly6C^hi^Ly6G- cells. Interestingly, spleens and livers of *L. donovani* infected STAT1KO mice showed profound defect in Ly6C monocyte accumulation (Fig. 4A,B). We then hypothesized that the defect in monocyte recruitment may account for the ability of STAT1 KO mice to efficiently control *L. donovani* infection. To test this hypothesis, we transferred bone marrow Ly6C^hi^ WT monocytes into STAT1 KO mice on 1 dpi. After 20 days of infection STAT1KO mice that received WT monocytes showed two times more parasites in the spleen than control STAT1 KO mice (Fig. 4C). However, we did not find differences in parasitic loads in the liver (data not shown). Splenocytes from STAT1 KO mice proliferated more than those from WT mice after *Leishmania* antigen re-stimulation. However, the proliferation of STAT1 KO splenocytes was slightly reduced by transferred WT iMOs (Fig. 4D). WT infected mice produced enhanced levels of IFNγ and IL-10 in splenocyte culture supernatants upon *L*. *donovani* Antigen (LdAg) stimulation. In contrast, the culture supernatants from STAT1 deficient mice showed reduced levels of IFNγ and IL-10 (Fig. 4G,H). Strikingly, we observed significantly higher levels of IL-17 production from STAT1KO mice compared to the WT mice (Fig. 4I). Interestingly, STAT1 KO mice that received WT monocytes recovered IL-10 production and showed impaired IL-17 production. In line with other reports showing a relationship between IL-17 levels and neutrophil accumulation we found significantly lower neutrophil frequencies in spleens and livers of STAT1 KO mice that received WT monocytes compared with their controls (Fig. 4E,F).

### Blocking iMO accumulation decreases parasitic loads in liver and spleen

**Figure 5:**
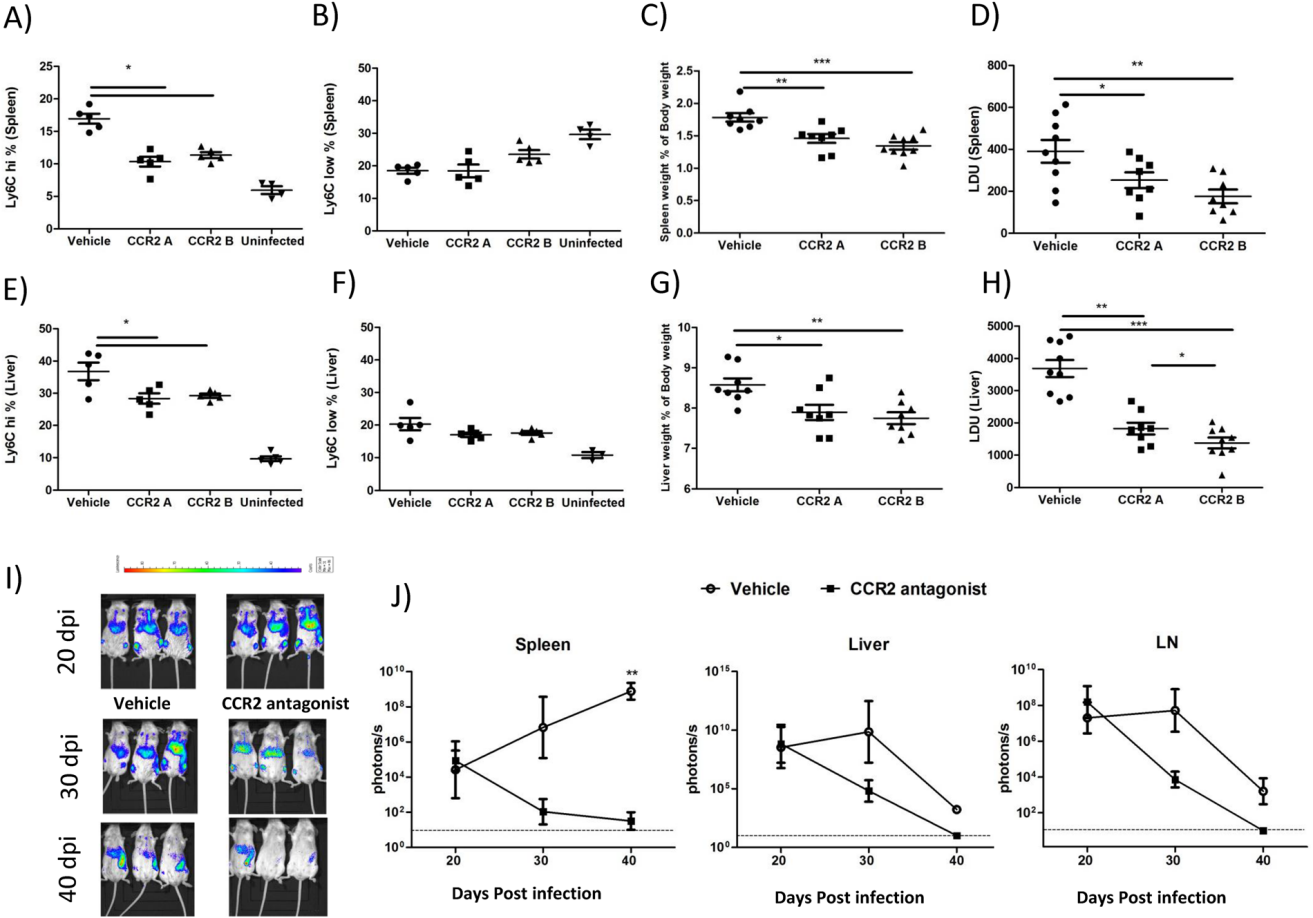
Administration of CCR2 antagonist reduced monocyte recruitment and parasitic loads. *L. donovani* infected mice were treated with CCR2 antagonist RS-504393, once daily from day 1 to day 30 pi (CCR2A) or once daily from day 20 to day 30 pi (CCR2B) respectively. Flow cytometry analysis of frequencies of Ly6C^hi^ cells in spleen (Fig. 5A) and liver (Fig. 5E). Flow cytometry analysis of frequencies of Ly6C^low^ cells in spleen (Fig. 5B) and liver (Fig. 5F). Data shown is from one of the three experiments (n = 4). Spleen weight (Fig. 5C) and liver weight (Fig. 5G) percentages of body weight were represented at 30 dpi. Combined data from two independent experiments N = 8, *p < 0.05 **p = 0.01 ***p = 0.001. (**I**,**J**) Representative images and Kinetics of Luciferin-expression *L. donovani* was analyzed by IVIS imaging system at the indicated time points. Data representative of the mean ± SEM of photons/second/cm^2^ (n = 3).

Our data showed that deficient recruitment of iMOs in STAT1KO mice improved resistance to VL. Monocytes egress from the bone marrow in a CCR2 dependent fashion^3^. In order to block monocyte migration into the infected tissues we administrated a CCR2 specific antagonist RS-504393, as was previously used in a fibrosis model^24^. We injected CCR2 antagonist once daily from day 1 to day 29 pi (CCR2A) and another group once daily from day 20 to day 30 pi (CCR2B). Both modes of RS-504393 administration effectively reduced the numbers of inflammatory monocytes in spleens and livers (Fig. 5A,E) without affecting the frequency of the CD115+ Ly6C^lo^ population (Fig. 5B,F). These findings strongly suggest that iMOs accumulated during VL were bone marrow derived and their migration was CCR2 dependent. Interestingly, mice which received RS-504393 presented less splenomegaly and hepatomegaly compared with control mice (Fig. 5C,G). Importantly, blocking iMOs accumulation by CCR2 antagonist resulted in significantly decreased parasitic loads in spleens and livers (Fig. 5D,H). A previous study by Sato *et al*.^25^ had found genetically resistant C57BL/6 mice lacking CCR2 develop similar parasitic burdens compared to WT upon *L. donovani* infection. Although Sato *et al*. did not examine the impact of CCR2 deficiency on iMOs recruitment to organs, these observations suggest that CCR2 and possibly iMOs recruited to the organs may not play a critical role in immunity against *L. donovani*. In an independent experiment by using luciferin expressing parasites, we also confirmed that CCR2 antagonist treatment successfully reduced the parasitic loads in livers, spleens and lymph nodes of *L. donovani* infected mice (Fig. 5I,J). Additionally, livers from mice treated with RS-504393 showed no granuloma formation irrespective of the period of RS-504393 administration (Supplementary Figure 1).

### CCR2 inhibition decreased IFNγ IL-10 double producers CD4+ T cells

**Figure 6:**
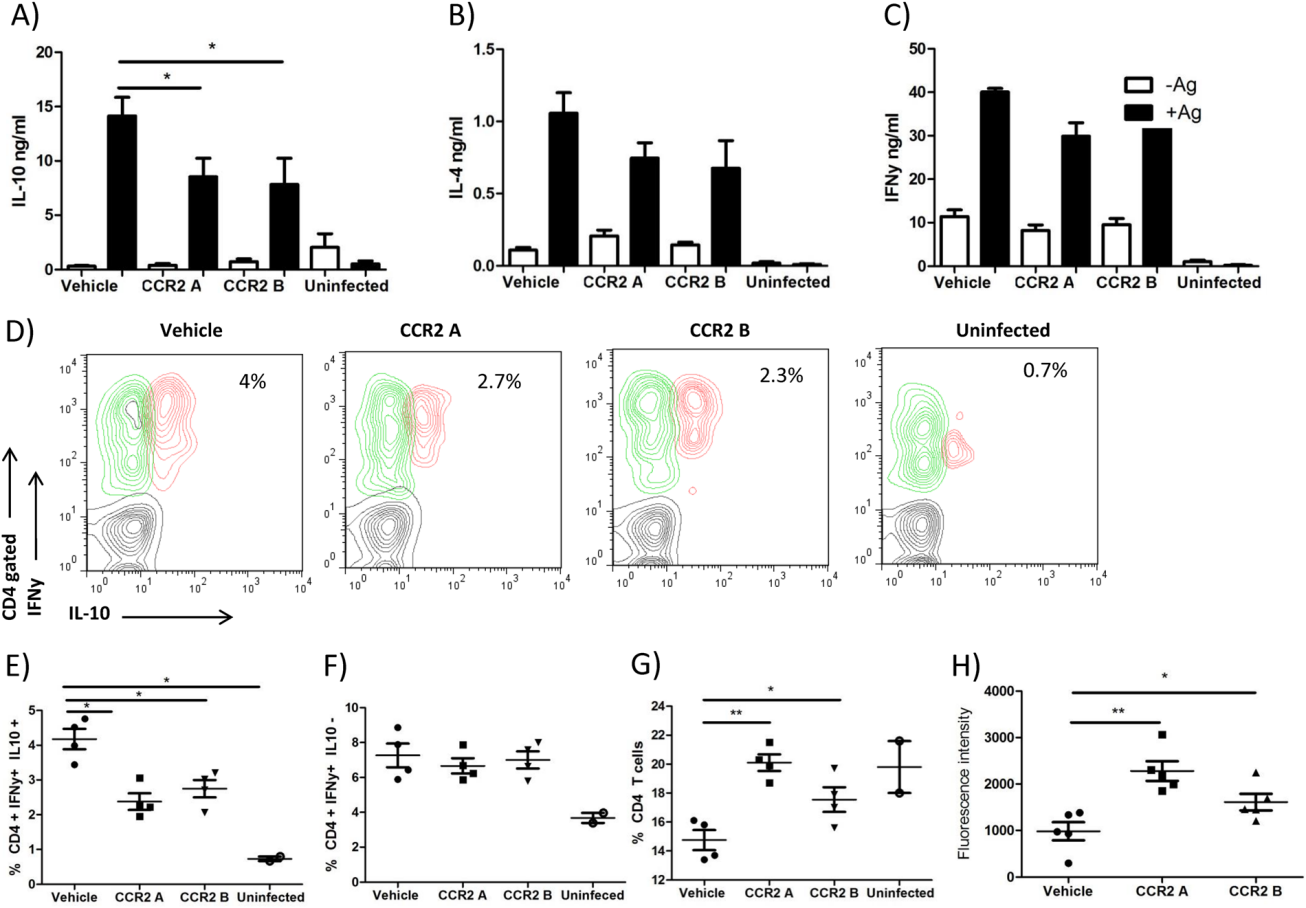
CCR2 antagonist reduced percentages of IFNγ+ IL10+ double producer CD4 cells. Splenocytes were harvested from *L. donovani* infected, CCR2A, CCR2B and vehicle treated mice at 30 dpi, re-stimulated with LdAg for 72 h, and release of cytokines was evaluated in the culture supernatants. (**A**) IFNγ, (**B**) IL-4 and (**C**) IL-10 release was evaluated by ELISA. (**D**) Representative dot plots showing IFNγ and/or IL10 production by CD4+ gated cells. Analysis of (**E**) frequencies of IFNγ IL-10 double producers CD4+ cells in the spleen, (**F**) IFNγ CD4+ producers cells, and (**G**) total CD4+ cells n = 4. (**H**) Proliferation analysis by alamar blue of splenocytes stimulated 72 h with *L. donovani* antigens. n = 5, *p < 0.05 **p = 0.01.

In order to investigate if the absence of iMOs affected the host immune response, we evaluated the cytokine profile of CCR2 inhibitor treated mice. We found that CCR2 inhibitor administration resulted in decreased IL-10 production by splenocytes independent of the time of administration of CCR2 antagonist (Fig. 6A). Interestingly, CCR2 inhibitor administration had no effects on IFNγ and slightly, but not significantly, decreased IL-4 production, leaving the Th1/Th2 response intact (Fig. 6C,B). IL-10 was significantly diminished when we administered CCR2 antagonist, correlating with disappearance of inflammatory monocytes (Fig. 5A,E). However, we did not observe up-regulation of IL-10 mRNA in inflammatory monocytes in the spleen (Fig. 2B), suggesting that iMO could be influencing other cells to produce IL-10. In order to evaluate the source of IL-10, we performed intracellular cytokine staining of splenocytes from chronically infected mice. We found a CD4+ T cell population positive for both IFNγ and IL-10 (Fig. 6D). This population has been reported to be responsible for non-healing lesions during *L. major* infection and with the progression of VL^26,27^. In our experiments, RS-504393 treated mice showed reduced numbers of IFNγ IL10 double producer CD4+ T cells (Fig. 6D,E) and correlated with decreased IL-10 production found in splenocyte supernatants (Fig. 6A). We also noticed that antigen specific splenocyte proliferation was enhanced in RS-504393 treated mice (Fig. 6H). In line with this, administration of CCR2 antagonist resulted in enhanced frequencies of CD4+ T cells (Fig. 6G) and comparable numbers of CD4+ IFNγ+ cells in the spleen (Fig. 6F). The splenocytes of CCR2 antagonist received mice also showed increased T cell proliferation upon *L. donovani* antigen stimulation (Fig. 6H).

## Discussion

In contrast with other infectious diseases, we found here that iMOs accumulated during VL and served as a continuous source of host cells for *L. donovani* parasite infection. Early recruited iMOs during VL did not show the classical activation profile, and lacked iNOS, TNFα, as well as expression markers for dendritic cells. iMOs from chronic infection demonstrated polarized profiles towards an inflammatory phenotype (iNOS) in the liver and an anti-inflammatory phenotype (arginase) in the spleen. This suggests that early contact with *L. donovani* is not able to trigger iMO activation and their polarization requires further help from the adaptive immune response. In mice, *L. donovani* profoundly infects the liver in the first 3–4 weeks and then the infection is resolved via an IL-12-IFNγ-iNOS dependent axis^18,28^. In the spleen the infection is chronic, progressive and does not resolve. This correlates with the high levels of iNOS we observed in liver iMOs which may partly account for their ability to kill the parasites and resolve liver infection^29^. On the other hand, higher arginase expression in splenic iMOs could potentially facilitate parasite growth and avoid its elimination.

It must be noted that blocking iMO accumulation was sufficient to avoid parasite establishment in the liver and spleen. This suggests that iMOs promote VL primarily as initiators of *L. donovani* establishment. Supporting this idea, we showed that STAT1 was required for iMO accumulation. This explains the resistant phenotype of STAT1 KO mice even in the absence of IFNγ signaling. It appears from this data that although STAT1 signaling is generally protective against VL, in some cases it can be detrimental, and could cause the accumulation of host reservoir cells for *L. donovani* infection^4^. STAT1 may participate in the differentiation of monocytes since IFNs play a role in monopoiesis^23^. IFNs are also important for the upregulation of integrins that facilitate iMO migration through the endothelia and tissues and by enhancing chemokine expression^30^. However, the exact mechanism of STAT1-mediated monocyte accumulation during VL requires further research.

iMOs require TLR or IFNγ stimulation to become activated. In contrast, the presence of IL-10 impairs their differentiation into TipDCs^31^. The ability of *L. donovani* to subvert TLR and IFNγ signaling and enhance IFNγ/IL10 producer CD4+ T cells can explain the inability of iMOs to become fully activated during VL. The iMO phenotype found in VL was in striking contrast to that found during cutaneous leishmaniasis, where iMOs differentiated into TipDCs with high nitric oxide production and leishmanicidal activity^32^. It has been proposed that the tissue microenvironment may control the immune response^33^, but parasites can also directly modify the behavior of innate myeloid cells^34^. In our study we still do not understand what causes the differences observed in monocyte polarization in the liver and spleen during VL, but it is possible that tissue-derived factors may play a role in regulating iMO activation.

Our results demonstrate as a proof of concept that blocking iMOs results in reduced parasitic loads during *L. donovani* infection. Importantly the absence of iMOs negatively affected the numbers of IFNγ/IL10+ CD4+ T cells which are also dependent on the presence of CD11c+ DCs and play a detrimental role during VL^27^. Interestingly, blocking iMO recruitment reduced granuloma formation. Although the formation of granulomas is important for *L. donovani* elimination from the liver, it has been shown that *L. donovani* elimination can be achieved in absence of granulomas^35^. The high expression of CXCL9 and S100A8/9 may account for the ability of iMOs to recruit neutrophils, monocytes and T cells into the infected site and shape granuloma formation.

In conclusion, our data provide evidence of a novel mechanism by which *L. donovani* successfully establishes infection in the host, which relies on the recruitment of iMOs. In this experimental VL model, these cells are critical for sustaining *L. donovani* infection by functioning as a host cell reservoir for the parasite, in addition to modulating the host immune response. However, it must be noted that there are significant differences between the well-established experimental VL model used in this study and human VL, which could have an impact on the significance of our results to human disease. For one thing, the intravenous injection of *L. donovani* amastigotes into mice contrasts with the natural sand-fly mediated cutaneous route of transmission of *L. donovani* promastigotes in humans. This obviously affects the nature of local and systemic immune responses involved in immunity to the human disease. Nevertheless, our novel findings underscore the importance of a unique immune cell subset that mediates the establishment of *L. donovani* infection in mice, which can potentially be exploited in the prevention and treatment of VL.

## Materials and Methods

### Animals

STAT1^−/−^ (C.129S-*Stat1*〈*tm1Dlv*〉) mice were generated as described previously^4^. 8–10 weeks old female BALB/c mice were purchased from Envigo (previously Harlan laboratories, Indianapolis, IN, USA). All experimental mice were maintained in vivarium at The Ohio State University in compliance with the guidelines of OSU ULAR. All experimental procedures were performed according to the approved experimental protocol 2010A0048-R2 by The Ohio State University institutional guidelines for animal research (OSU-IACUC).

### Parasites and infection protocol

*Leishmania donovani* (LV82 strain) amastigotes were isolated from the spleen of previously infected Golden Syrian hamsters. Red fluorescent protein expressing DsRed2 transgenic *L. donovani* LV82 parasites were obtained from infected stock mice as described previously^36^. All experimental mice were infected with 10^7^ *L. donovani* LV82 or DsRed2 LV82 amastigotes by tail vein injection. Groups constituted of three or five mice and were sacrificed at different time points post-infection for further analysis.

### Parasite burden calculation

At different time points during the infection, livers and spleens were harvested, weighed, and impression smears were taken. Smears were stained with Giemsa stain (Sigma-Aldrich, St. Louis, MO, USA). Parasite loads were counted under a microscope by a certified pathologist. Parasitic loads were expressed as *Leishmania donovani* Units (LDU) = Number of amastigotes per 1000 nucleated cells × organ weight (grams).

### Treatment of mice

To evaluate the role of CCR2 inflammatory monocytes, we administered a CCR2 antagonist RS-504393 (R&D systems, Minneapolis, MN, USA) intraperitoneally. One group of mice received RS-504393 (2 mg/kg i.p.) one day after infection daily for 29 days (CCR2 A), second group received RS-504393 (2 mg/kg i.p) daily from days 20–30 dpi (CCR2 B). Control mice were injected with PBS intraperitoneally.

### Cytokine ELISA and Alamar blue proliferation assay

Splenocytes isolated from WT and *STAT1*−/− mice were plated at a concentration of 0.5 × 10^6^ cells per well in quadruplicates in 96-well tissue culture plates (Thermo-Fisher Scientific, Waltham, MA, USA). Cells were then stimulated with 20 ug/ml of *L. donovani* antigen (LdAg) prepared by freeze-thawing *L. donovani* promastigotes. Supernatants were collected after 72 h of incubation and analyzed for the production of IFN-γ, IL-4, IL-6, IL-10, IL-13 and IL-17 by ELISA (all capture and detection antibodies were purchased from Biolegend, San Diego, CA, USA) and recombinant mouse standards were purchased from BD Biosciences (San Jose, CA, USA). For proliferation assays 10% Alamar blue (Life technologies, Carlsbad, CA) was added 48h after stimuli and readings were taken after additional 18 hours of incubation. Data represent fluorescence units calculated by subtracting the fluorescence of unstimulated controls versus *L. donovani* stimulated cells. ELISA plates were read at an absorbance of 405 nm, and for Alamar blue, plates were read at 570 and 600nm using Spectramax M3 microplate reader (Molecular Devices LLC, Sunnyvale, CA, USA) and concentrations were determined by Softmax Pro software (Molecular Devices LLC, Sunnyvale, CA, USA).

### Flow cytometry

Single cell suspensions from liver and spleen were collected gently extruding the tissues through a 70 uM cell strainer (BD Biosciences, San Jose, CA, USA). Blood was recovered from tail vein bleeding. Red blood cells were lysed with ACK lysis solution. One million cells were blocked with mouse serum and stained with different cocktail antibodies or their respective isotype controls for 20 minutes at 4 °C. Ly6C APC, Ly6G PE, CD11b FITC, CD62L PercP, MHCII PE, F480 PercypCy7, CD115 PercP, and iNOS PE were purchased from Biolegend (San Diego, CA, USA), and CCR2 Alexa 700 or FITC, Arginase APC from R&D systems (Minneapolis, MN, USA). For intracellular detection of iNOS and arginase, cell membrane was stained first and then fixed with 2% paraformaldehyde for 10 minutes. Cell permeabilization was carried out using Biolegend permeabilization buffer followed by 45-minute incubation with anti iNOS and anti-arginase antibodies. Flow cytometry was performed using by BD FACSCalibur (San Jose, CA, USA) or Flowsight AMNIS (Seattle, WA, USA) cytometers. Cell sorting was performed using a BD FACS Aria (San Jose, CA, USA). Sorted Ly6C^hi^ Ly6G-CD11b+ cells were isolated from bone marrow of BALB/c naïve mice, and 1 × 10^6^ Ly6C+ Ly6G-CD11b+ cells were transferred i.v. into STAT1 KO mice one day before *Leishmania donovani* infection. For monocyte and neutrophil functional assays, Ly6C^hi^ Ly6G- CD11b+ or Ly6C+ Ly6G+ CD11b+ expressing cells were isolated from the spleens of naïve or infected mice. Data was analyzed using Flow jo software (FLOWJO, LLC, Ashland, OR, USA).

### Real Time PCR

Total RNA was extracted from both the spleens and livers of experimental mice by TRIzol extraction method (purchased from Life technologies (Carlsbad, CA)), making sure to minimize any contaminating genomic DNA material. cDNA was prepared by iScript reverse transcriptase and RT PCR reactions were done by using IQ SYBR green super mix and a CFX 96 RT-PCR cycler (purchased from Bio-Rad, Hercules, CA, USA). Pre-validated primers were selected from the Primer bank website (http://pga.mgh.harvard.edu/primerbank) and are listed in Supplementary Table 1. Samples were run in technical triplicates, and appropriate positive and negative controls were included for each RT-PCR experiment. Data obtained were normalized using the housekeeping gene β-actin and presented as the fold induction over uninfected WT mice.

### Microarray

Five ng of total RNA was amplified using the NuGen Ovation Pico WTA System V2 and labeled by using the NuGen Encore Biotin Module (San Carlos, CA, USA). The samples were then hybridized onto the Mouse Transcriptome arrays (MTA) for 16 hours at 45 °C. The samples were then washed and stained on the Affymetrix Fluidics 450 system according to manufacturer protocols and scanned on the Affymetrix GeneChip scanner 3000 7G (Sunnyvale, CA, USA).

### Statistical analysis

Student’s unpaired *t*-test was used to determine statistical significance of differences in the values. A value of *p* < 0.05 was considered significant.

## Electronic supplementary material

Supplementary figure
