# Supplementary material for: Ly6Chi inflammatory monocytes promote susceptibility to Leishmania donovani infection
Source: Sci Rep. 2017 Oct 31;7:14693. doi: 10.1038/s41598-017-14935-3 (PMC5665970; doi:10.1038/s41598-017-14935-3)
Supplement: Supplementary file 1 — Supplementary figure [file 41598_2017_14935_MOESM1_ESM.pdf]

Ly6C^{hi} inflammatory monocytes promote susceptibility to *Leishmania donovani* infection

Cesar Terrazas , Sanjay Varikuti , Steve Oghumu , Heidi M Steinkamp , Nurittin Ardic , Jennifer Kimble , Hira Nakhasi , Abhay Satoskar.

Supplementary figure 1

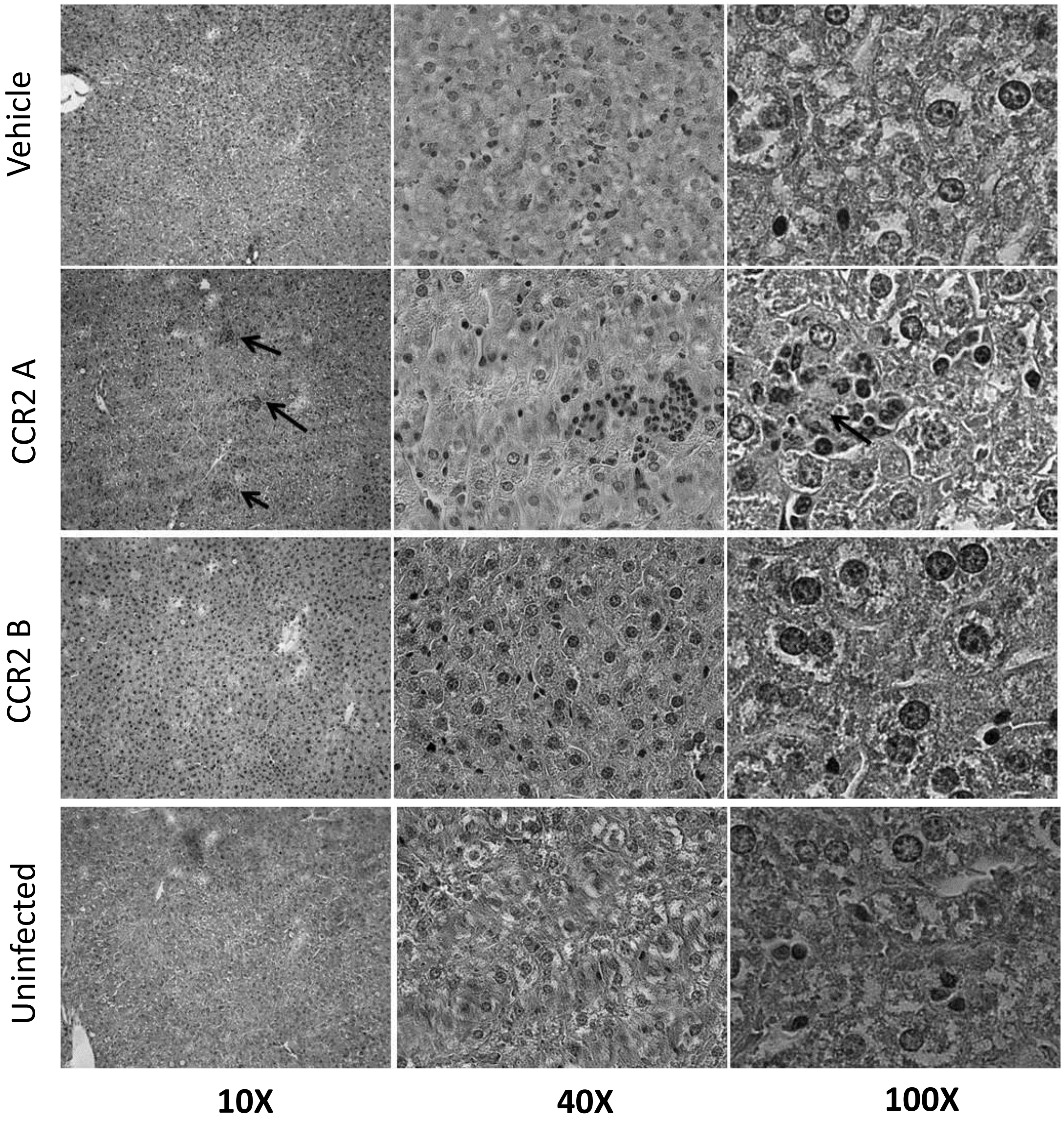

Supplementary figure 1. CCR2 antagonist administration reduces liver pathology and granuloma formation during VL. H & E staining of liver sections recovered from infected mice after 30 dpi. Mice were infected i.v. with L donovani amastigotes; vehicle control received PBS

i.p. daily starting 1 dpi. CCR2 A group received CCR2 antagonist starting from day 1 to day 30dpi. CCR2 B group received CCR2 antagonist from day 20 to day 30dpi.

Supplementary table: 1

Primer sequences used in the study.

Primer Name	Primer sequence: 5’-3’
Arginase Forward primer	TCC TCC ACG GGC AAA TTC C
Arginase Reverse primer	GCT GGA CCA TAT TCC ACT CCT A
INOS Forward primer	GTT CTC AGC CCA ACA ATA CAA GA
INOS Reverse primer	GTG GAC GGG TCG ATG TCA C
IL27-P8 Forward primer	CTC CTG GGA ACC TTT GGG C
IL27-P8 Reverse primer	CGT CCC TTT TGT GTC CCC C
IL-10 Forward primer	GAC TTT AAG GGT TAC CTG GGT TG
IL-10 Reverse primer	TCA CAT GCG CCT TGA TGT CTG
TNF-A Forward primer	CCT CTC TCT AAT CAG CCC TCT G
TNF-A Reverse primer	GAG GAC CTG GGA GTA GAT GAG
TGFB Forward primer	GGC CAG ATC CTG TCC AAG C
TGFB Reverse primer	GTG GGT TTC CAC CAT TAG CAC
COX 2 Forward primer	CAC CCT GAC ATA GAC AGT GAA AG
COX 2 Reverse primer	CTG GGT CAC GTT GGA TGA GG
CXCL9 Forward primer	GGAGTTCGAGGAACCCTAGTG
CXCL9 Reverse primer	GGGATTTGTAGTGGATCGTGC
IRF-1 Forward primer	ATG CCA ATC ACT CGA ATG CG
IRF-1 Reverse primer	CCT GCT TTG TAT CGG CCT GT
IRG1 Forward primer	ACT CAG ACT AAG CCC CTT CAT
IRG1 Reverse primer	GGA AGC TCT TAA AGG CCA CAT
S100A8 Forward primer	AAA TCA CCA TGC CCT CTA CAA G
S100A8 Reverse primer	CCC ACT TTT ATC ACC ATC GCA A
S100A9 Forward primer	ATA CTC TAG GAA GGA AGG ACA CC
S100A9 Reverse primer	TCC ATG ATG TCA TTT ATG AGG GC
SERPIN 3 GA Forward primer	CTT CCC AAC GGC TGG AAT CTA

SERPIN 3 GA Reverse primer	ACT GTC CAA TCA GGC ATA GCG
beta-Actin- Forward primer	CTG GCA CCA CAC CTT CTA CA
beta-Actin- Reverse primer	CTT TTC ACG GTT GGC CTT AG
